# Neurosensory sequelae assessed by thermal and vibrotactile perception thresholds after local cold injury

**DOI:** 10.3402/ijch.v73.23540

**Published:** 2014-02-17

**Authors:** Daniel Carlsson, Lage Burström, Victoria Heldestad Lilliesköld, Tohr Nilsson, Erik Nordh, Jens Wahlström

**Affiliations:** 1 Occupational and Environmental Medicine, Department of Public Health and Clinical Medicine, Umeå University, Umeå, Sweden; 2 Department of Occupational and Environmental Medicine, Sundsvall Hospital, Sundsvall, Sweden; 3 Division of Clinical Neurophysiology, Umeå University, Umeå, Sweden

**Keywords:** case series, neurovascular, quantitative sensory testing, military, frostbite, Sweden

## Abstract

**Background:**

Local freezing cold injuries are common in the north and sequelae to cold injury can persist many years. Quantitative sensory testing (QST) can be used to assess neurosensory symptoms but has previously not been used on cold injury patients.

**Objective:**

To evaluate neurosensory sequelae after local freezing cold injury by thermal and vibrotactile perception thresholds and by symptom descriptions.

**Design:**

Fifteen patients with a local freezing cold injury in the hands or feet, acquired during military training, were studied with QST by assessment of vibrotactile (VPT), warmth (WPT) and cold (CPT) perception thresholds 4 months post-injury. In addition, a follow-up questionnaire, focusing on neurovascular symptoms, was completed 4 months and 4 years post-injury.

**Results:**

QST demonstrated abnormal findings in one or both affected hands for VPT in 6 patients, for WPT in 4 patients and for CPT in 1 patient. In the feet, QST was abnormal for VPT in one or both affected feet in 8 patients, for WPT in 6 patients and for CPT in 4 patients. Freezing cold injury related symptoms, e.g. pain/discomfort when exposed to cold, cold sensation and white fingers were common at 4 months and persisted 4 years after the initial injury.

**Conclusions:**

Neurosensory sequelae after local freezing cold injury, in terms of abnormal thermal and/or vibration perception thresholds, may last at least 4 months after the initial injury. Symptoms such as pain/discomfort at cold exposure, cold sensations and white fingers may persist at least 4 years after the initial injury.

Civilians at risk of sustaining injuries from the cold are homeless persons, those involved in outdoor leisure activities in a cold climate and those whose work requires them to spend time in freezing temperatures (1). Exposure to cold at work is common in the Nordic countries. Official statistics from 2011 show that 22% of all men and 10% of all women in Sweden are exposed to cold at work for more than 25% of their working hours (2). The lifetime prevalence of severe frostbite in Finland has been estimated at 11% of the entire population, with a higher figure among those in occupational groups exposed to cold (1). Local cold injuries can be divided into freezing cold injuries, occurring at temperatures below 0°C, and non-freezing cold injuries which occur after prolonged exposure to temperatures just above 0°C, together with wet conditions and local pressure (3). The pathophysiology and symptoms in the acute and sub-acute phases are well described in the literature regarding both freezing- and non-freezing cold injuries (1) and a number of studies have shown that sequelae to cold injury can persist for many years (4–7). Several studies report remaining neurological symptoms such as cold sensitivity, hypoesthesia and pain in the affected area (8). The underlying pathophysiological mechanisms to these complex phenomena are not clearly understood. To indirectly assess these kinds of neurological symptoms a non-invasive psychophysical semi-objective assessment of detection thresholds for warmth, cold and vibration stimuli, denoted “quantitative sensory testing” (QST), can be used (9). To our knowledge, the possible neurosensory sequelae of freezing cold injuries to the hands and feet, have not earlier been investigated using QST.

The aim of this study was to evaluate neurosensory sequelae after local freezing cold injury by thermal and vibrotactile (VPT) perception thresholds and by symptom descriptions.

## Methods

While doing winter training in the north of Sweden 2007, a military platoon of approximately 80 conscripts spent several prolonged exercise periods in an extremely cold climate, performing various cold-exposure activities, such as full body winter water immersion. In one particular exercise the group did not cope satisfactorily with the cold climate, resulting in several cold injuries among the conscripts. Thirty-four conscripts reported physical problems to the on-site physician in connection with the exercise. In June the same year, 4 months post-injury, they were all interviewed and examined by the physician in our research team. In addition to the physical examination, QST of thermal and vibration thresholds in the hands and feet was performed and a self-administered questionnaire was completed. Four years later, in November 2010, the same group was asked to complete the same questionnaire, with a few additional questions.

The Regional Ethical Review Board at the medical faculty at Umeå University, Sweden approved the study.

### Patients

The inclusion or exclusion of patients in this study was based on information obtained from the conscripts’ medical histories. The inclusion criteria were: a) The patient must have developed a freezing cold injury in at least one of his hands or feet during his military training, and b) participation in the medical interview, the physical examination and in all QST examinations. The inclusion process was performed in 2 steps In the first step, 14 from a total of 34 patients were excluded because their reported cold injury was not located in their hands or feet or because their case record entries did not contain sufficient information to ensure that the patient had *clearly* developed a freezing cold injury in at least one of his hands or feet during his military training. In the second step, 5 patients out of the remaining 20 were excluded, as they had not participated in all QST examinations.

Fifteen patients with a median age at the time of injury of 20 years (range 19–21 years) remained in the study for further analyses, 10 had a freezing cold injury to one or both hands and 9 had a freezing cold injury to one or both feet (Table I). The cold injuries were not classified according to the conventional system due to lack of precise information from medical records regarding the acute stages of the injuries. However, no information in the medical history indicated cold injuries severe enough to fulfil the criteria for a third or fourth degree cold injury (tissue necroses, blue/grey discolouring, etc.). Hence, it is most likely that all of the cold injuries were first or second degree, as indicated by notations of vesicles with clear liquid, occasional skin desquamation, erythema, oedema, and hyperaemia.

**Table I:** Site of injury and participation 4 months (4 m) and 4 years (4 y) post-injury for all patients (n=15)

						Participation 4 m						
							
		Site of cold injury					Vibrotactile			Thermotactile		
						
		Hand		Foot			Hands		Feet	Hands	Feet	Participation 4 y
								
ID	Age	R^1^	L^2^	R^1^	L^2^	Q^3^	Dig 2	Dig 5	Dig 1	Dig 2+3	D foot^4^	Q^3^
1	21	●	●	○	○	●	●	●	●	●	●	●
2	21	○	○	○	●	●	●	●	●	●	●	●
3	21	●	●	○	○	●	●	●	●	●	●	●
4	20	●	●	○	●	●	●	●	●	●	●	●
5	21	●	●	○	○	●	●	●	●	●	●	●
6	19	●	●	●	●	○	●	●	●	●	●	●
7	20	○	○	●	○	○	●	●	●	●	●	●
8	20	●	●	●	●	●	●	●	●	●	●	○
9	20	●	●	○	○	●	●	●	●	●	●	●
10	19	●	●	○	○	●	●	●	●	●	●	●
11	19	○	○	○	●	●	●	●	●	●	●	●
12	21	●	●	●	●	●	●	●	●	●	●	●
13	20	○	○	●	○	●	●	●	●	●	●	●
14	20	○	○	●	●	○	●	●	●	●	●	●
15	20	●	○	○	○	●	●	●	●	●	●	○

Solid circle indicates that the patient has a freezing cold injury at the specific site or participated in the questionnaire or assessment specified.

1 Right

2 left

3 questionnaire

4 dorsal foot.

### Exposure

During the winter of 2006–2007, the weather station in Arvidsjaur, where the patients were stationed for their military training, recorded 46 days with temperatures below −10°C. The lowest temperature recorded was −28°C according to official data from the National Swedish Meteorological and Hydrological Institute (10). Even though the ambient temperature were mostly below zero, giving low outdoor humidity and no rain, outdoor activities with frequent contact to snow and ice as well as sweating causes clothes to become wet, primarily gloves, shoes and socks. Wind wet clothes and direct contact with cold surfaces may have further aggravated the effect of the cold.

### Physical examination and medical history

A retrospective medical history was taken and musculoskeletal, neurological and vascular examinations were performed. In this study only the anamnestic information from the medical history was used to determine whether or not a freezing cold injury had been present. The medical interview focused on earlier or current physical problems possibly caused by exposure to cold during military training.

### Thermal and vibrotactile perception threshold measurements

Thermal perception testing was performed bilaterally at the palmar surface of the distal phalanges of the second and third fingers, and on the dorsal surface of the foot arch. Vibrotactile testing was performed bilaterally at 3 sites; at the palmar surface of the distal phalanges of the second and third fingers in the hand and at the plantar surface of the distal phalange of the first toe on the foot. Prior to the measurements, the skin temperature was measured to ensure a minimum temperature of 31°C. If the temperatures were lower, the hand and/or foot were heated by immersion in warm water.

Thermal cold (CPT) and warm (WPT) perception thresholds were assessed according to the method-of-limits (11). Heating and cooling stimuli were delivered with a 2.5×5.0 cm stimulation probe of Peltier-type (Thermotest^®^, v. 01-S, Somedic AB, Hörby, Sweden). During the testing, the probe was held manually to obtain maximum skin contact, with care taken to exert a similar pressure on all patients and at all test sites. Ten cold stimuli were consecutively followed by 10 warm stimuli, and the thermal thresholds at the tested point were defined by the average of the individual cold and warm threshold reported by the patient for each stimulus. The test probe baseline temperature was set to 32°C. Maximum probe temperature was set to 52°C and minimum to 10°C, outside these limits the sensory testing was aborted for reasons of patient safety, in accordance with prevailing mandatory hospital safety regulations. The rate of temperature change was set to 1°C/s in the test phase and 3°C/s when the probe returned to the baseline temperature. At the testing, the patient was instructed to press a button as soon as thermal sensations were perceived, thereby causing a temperature read-out by the controlling computer and a return to the baseline probe temperature. The thermotactile outcome variable was the difference in degrees Celsius from baseline temperature to detection temperature.

The vibrotactile perception thresholds (VPT) were assessed with a VibroSense Meter (VibroSense Dynamics AB, Malmö). VPT were determined at 4 frequencies: 8, 32, 125 and 500 Hz according to the von Békésy method, in a manner compliant with the stipulations in the ISO 13091-1 (12). The patient was sitting comfortably at a table with the forearm and hand/foot supported on the test table and the whole of the distal phalanx pressed to a small circular metal probe at a predefined force. The magnitude of the probe vibration increased gradually until perceived by the patient, who then pressed a button. This caused the vibration magnitude to decrease, until the patient could no longer perceive it. At this time, the patient released the button and the vibration magnitude subsequently increased until the next detection. The VPT was presented as a sensibility index, defined as the ratio between the patient's determined VPT and the VPT of a reference population of the corresponding age. A sensibility index of 1.0 indicates a VPT equivalent to those of the reference population and a lower value indicates reduced sensibility to vibration stimuli (13).

### Pain and neurovascular symptoms

Pain and neurovascular symptoms were assessed by means of a questionnaire in conjunction with the other examinations, 4 months post-injury, and at the 4-year follow-up a questionnaire was distributed by post to the patients. The 6 patients (ID 1, 3, 7, 14, 23 and 27) who had a freezing cold injury to the hands and participated after both 4 months and 4 years were included in the analysis regarding pain and neurovascular symptoms in the hands. The 6 patients (ID 2, 7, 26, 27, 30 and 31) who had a freezing cold injury to the feet and participated after both 4 months and 4 years were included in the analysis regarding pain in the feet. For the questions regarding neurovascular symptoms in the feet, ID 16 and 18 were also included in the analysis since participation after 4 months was not required (n=8) (Table I). Pain ratings were made on an 11-point scale, with 0=“no pain” and 10=“pain as bad as it could be” (14). The perceived pain intensity was assessed by a question asking for the “average pain in the hands/fingers and feet/toes during the past month.” The questions regarding neurovascular symptoms were: a) “Do you have cold sensations in your hands/fingers?”; b) “Do you have white (pale) fingers of the type that appear when exposed to damp and cold weather?” (15); c) “Do you experience pain/discomfort when exposed to cold?”. Neurovascular symptoms were assessed from 4-categories response scale, comprising “no,” “insignificant,” “somewhat” and “quite a lot.” The questions were identical at 4 months and 4 years, with the exception that reports of symptoms in the feet were not included in the questionnaire at 4 months.

### Reference values

As reference values for QST, unpublished data derived from 80 male military conscripts doing their military training in Arvidsjaur 2009–2010 were used. The subjects in the reference group were enrolled in the same military training and had the same age range as the patients included in this study. The reference values were gathered during the reference conscripts’ first week of duty, before any exposure to cold or military exercises, using the same equipment for QST as in this study. However, as no reference values for WPT and CPT in the feet were available for this control group, reference values from 15 age-matched healthy male volunteers were used instead for WPT and CPT (mean 26±5, median 27, range 16–32 years). The thermal QST testing procedures in the hands also differed between patients and the reference group in 1 aspect; in the reference group only the palmar surface of the distal phalange of dig 2 was in contact with the probe, while amongst the study patients, the palmar surfaces of the distal phalanges of both dig 2 *and* 3 were touching the probe. For the present study a test value that falls below 2 standard deviations from the particular mean reference value adopted, in WPT and CPT, and exceeds 2 standard deviations from the mean reference value in VPT, was considered abnormally impaired and is hereafter denoted “abnormal.”

## Results

### Thermal and vibrotactile perception threshold measurements

Four months post-injury, 10 patients had a local freezing cold injury to their hands; 9 patients bilaterally and 1 unilaterally. We found abnormal values in one or both affected hands for VPT in 6 patients, for WPT in 4 patients and for CPT in 1 patient. Four patients identified with freezing cold injury to the hands did not have any abnormal findings in the hands (Table II).

**Table II:** Vibrotactile perception thresholds for digs 2 and 5 of the right and left hands and thermal perception thresholds for the distal phalanges of digs 2 and 3

	Vibration threshold^1^				Thermal threshold^2^			
		
	Dig 2		Dig 5		Warm		Cold	
				
ID	Right (%)	Left (%)	Right (%)	Left (%)	Right (°C)	Left (°C)	Right (°C)	Left (°C)
1	**0.77**	**0.27**	**0.56**	**0.04**	**7.0**	**13.7**	4.4	3.1
2	0.94	**0.80**	**0.80**	**0.68**	2.7	2.9	1.6	1.7
3	0.95	0.98	0.90	0.94	2.2	4.1	1.4	2.4
4	**0.81**	**0.77**	**0.63**	**0.75**	2.7	2.9	1.4	1.4
5	1.17	1.23	1.06	1.06	**7.2**	4.7	3.8	3.0
6	**0.76**	**0.68**	**0.47**	**0.62**	4.3	3.4	2.7	2.3
7	1.12	1.29	1.23	1.31	2.8	2.2	2.1	1.9
8	**0.55**	**0.55**	**0.56**	**0.57**	**5.6**	**6.7**	2.3	**6.6**
9	0.93	0.94	**0.78**	**0.74**	5.1	3.5	4.0	3.4
10	1.07	1.05	0.97	0.95	3.1	4.2	2.2	2.0
11	0.89	1.08	**0.83**	0.95	4.2	3.0	2.0	1.5
12	**0.73**	**0.90**	0.93	**0.82**	4.2	**8.3**	2.8	4.6
13	1.22	1.30	1.25	1.00	3.1	1.7	1.5	1.5
14	1.03	1.14	1.09	1.11	1.9	1.7	1.4	0.9
15	1.06	1.15	1.07	1.08	2.0	2.4	1.6	1.4

Numbers with white background indicate a test result from a hand with a freezing cold injury. Bold numbers indicate a test value that differs >2SD from the mean reference value.

1 Results presented as a sensibility index for all frequencies combined (8, 32, 125 and 500 Hz). A lower value indicates reduced sensibility to vibration stimuli.

2 Values presented are the difference in degrees Celsius from baseline temperature to detection temperature. A higher temperature value indicates reduced sensibility.

Four months post-injury, 9 patients had a local freezing cold injury to their feet; 4 patients bilaterally and 5 patients unilaterally. We found abnormal values in one or both affected feet for VPT in 8 patients, for WPT in 6 patients and for CPT in 4 patients. Two of the eight patients with abnormal VPT and 1 of the 6 with abnormal WPT had an abnormal value in only 1 foot even though both feet had sustained freezing cold injuries. Only one patient who identified with cold injury to the foot did not have any abnormal values (Table III).

**Table III:** Vibrotactile perception thresholds for the first toe of the right and left feet and thermal perception thresholds for the dorsal arc of the feet

	Vibration threshold^1^		Thermal threshold^8^			
		
	First toe		Warm		Cold	
			
ID	Right (%)	Left (%)	Right (°C)	Left (°C)	Right (°C)	Left (°C)
1	**0.29**	**0.28**	**9.1**	**11.5**	**2.9**	1.9
2	**0.24**	**0.32**	**11.7**	**11.5**	1.1	1.0
3	**0.31**	**0.05**	**11.1**	**12.3**	**8.7**	**2.8**
4	**0.34**	**0.27**	**6.9**	5.0	1.5	1.0
5	0.94	0.98	**11.0**	**11.1**	1.2	**6.3**
6	**0.28**	**0.33**	**9.8**	**9.5**	**2.8**	**2.3**
7	0.96	0.54	5.5	**6.0**	1.5	0.8
8	**0.05**	**0.09**	**9.0**	**12.3**	**9.4**	**8.7**
9	0.57	**0.42**	**13.4**	**12.7**	**5.7**	**2.8**
10	0.59	**0.34**	**9.5**	**7.6**	1.1	2.0
11	0.53	**0.48**	**9.3**	**10.2**	1.2	**5.7**
12	**0.45**	0.56	**8.3**	**7.2**	**2.3**	**4.5**
13	**0.44**	0.52	5.1	**5.9**	1.1	**2.2**
14	0.57	**0.28**	**6.2**	3.8	1.0	1.1
15	1.05	1.03	3.6	4.8	1.5	0.9

Numbers with a white background indicate a test result from a foot with a freezing cold injury. Bold numbers indicate a test value that differs >2SD from the mean reference value.

1 Results presented as a sensibility index for all frequencies combined (8, 32, 125 and 500 Hz). A lower value indicates reduced sensibility to vibration stimuli.

2 Values presented are the difference in degrees Celsius from baseline temperature to detection temperature. A higher temperature value indicates reduced sensibility.

### Pain and neurovascular symptoms

The group mean pain rating (range in parenthesis) was 1.6 (0–4) after 4 months and 1.6 (0–7) after 4 years in the hands together with 2.6 (1–4) after 4 months and 2.5 (0–8) after 4 years in the feet. Among those who completed both questionnaires, the majority reported a decrease in pain after 4 years. One patient reported increased pain in both hands and feet, one reported increased pain in the hands, and one reported increased pain in the feet. However, the levels of pain and the magnitude of the changes in pain were in general relatively low.

**Fig. 1:**
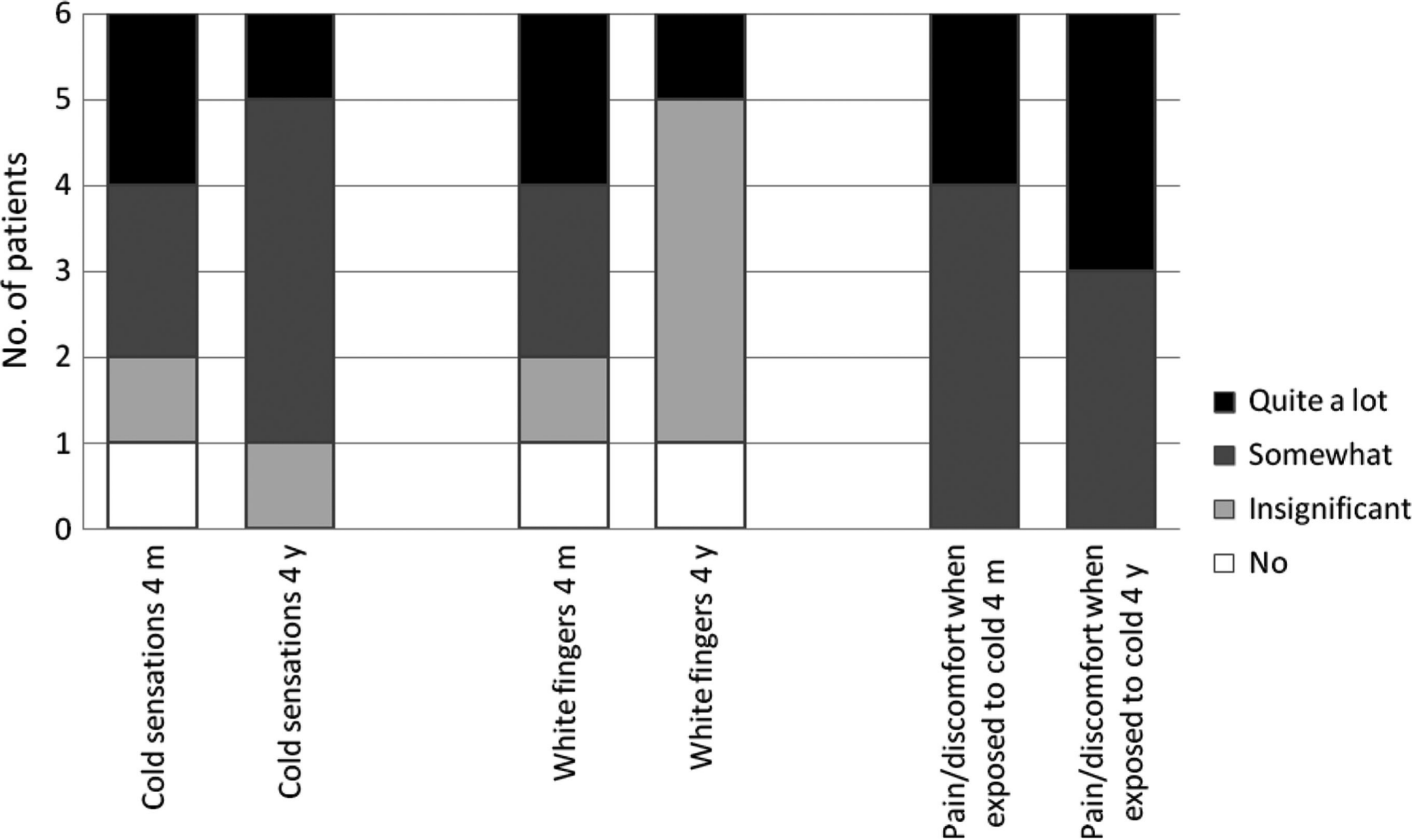
Neurovascular symptoms in the hands (n=6). Reported rate of neurovascular symptoms; cold sensation, white toes and pain/discomfort when exposed to cold, among patients with freezing cold injury to the hands 4 months (4 m) and 4 years (4 y) post-injury.

**Fig. 2:**
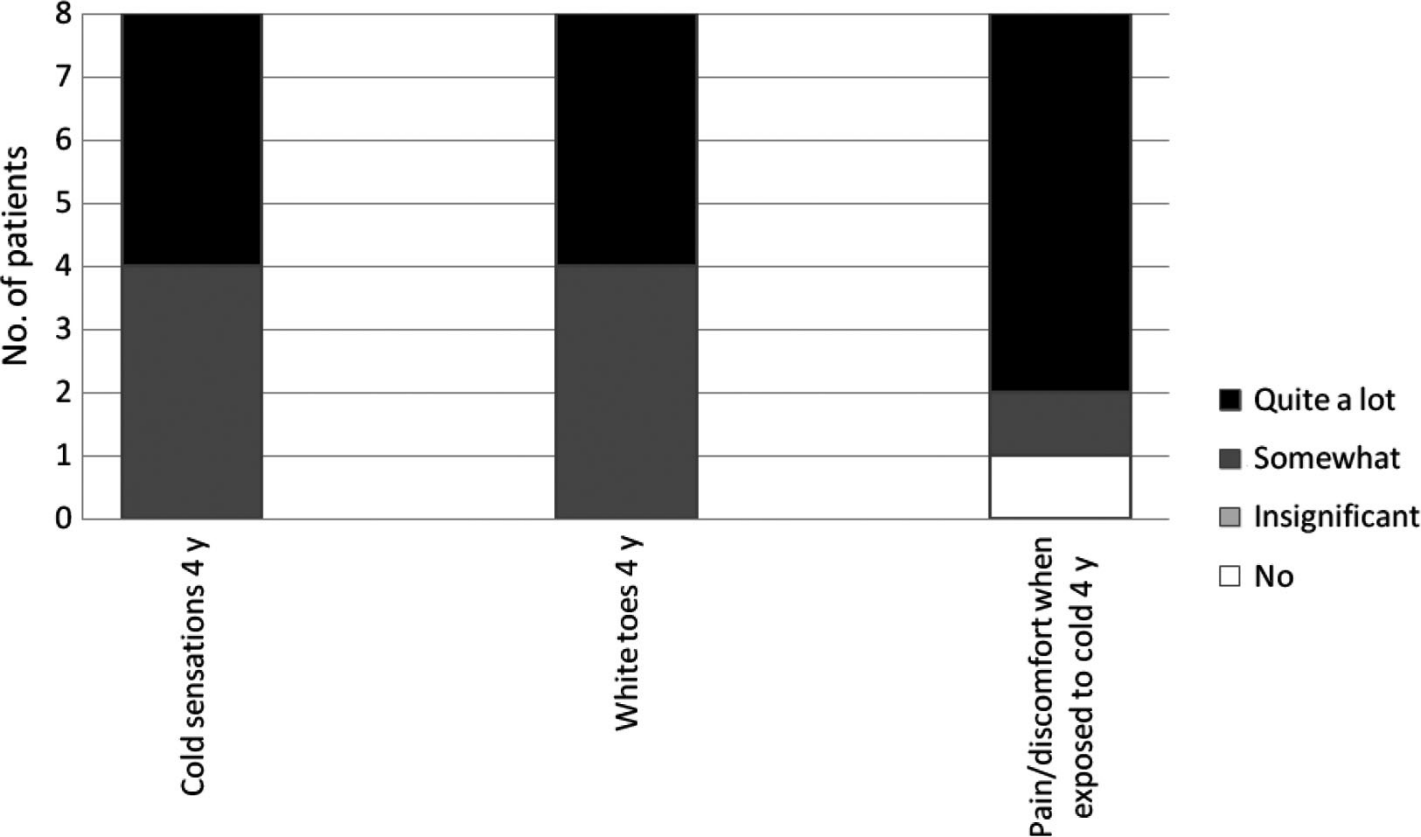
Neurovascular symptoms in the feet (n=8). Reported rate of neurovascular symptoms; cold sensation, white toes and pain/discomfort when exposed to cold, among patients with freezing cold injury to the feet 4 years post-injury (4 y).

All patients included in the analysis (n=6) reported neurovascular symptoms to some extent in the affected hand 4 months post-injury, and at group level these symptoms had persisted or even slightly increased after 4 years (Fig. 1). The 4-month questionnaire did not address the question of neurovascular symptoms in the feet, but in the 4-year follow-up, all included patients (n=8) reported neurovascular symptoms to some extent. Four out of eight patients reported the most severe rating, which was that they experienced “quite a lot” of “cold sensation” and the same was true for “white toes.” For “pain/discomfort when exposed to cold,” 6 out of 8 reported “quite a lot” (Fig. 2).

## Discussion

The main findings of the present report was that abnormal thermal and/or VPT were found in 11 out of 15 patients exposed to a local freezing cold injury, and that neurovascular symptoms, such as pain/discomfort at cold exposure, cold sensations and/or white fingers, can persist at least 4 years after the initial injury.

The results from the follow-up questionnaire suggest that neurovascular symptoms such as pain/discomfort at cold exposure, cold sensations and white fingers, can persist at least 4 years after the initial injury. The findings of such phenomena after cold injury are in line with the review covering international studies on this topic up to 2000 by Hassi and Makinen (8). That sequelae from cold injuries can persist many years after the initial injury is sustained is well known, and has been shown in numerous studies (4–7). A follow-up study by Ervasti (6) even shows that symptoms following severe frostbite in the lower extremities can persist after 30 years which indicates that the sequelae could be chronic and life-long lasting. Local freezing cold injuries do not have to be severe to cause long-term sequelae. Most of the injuries described by our patients were likely to be superficial first or second degree cold injuries and our study shows that even such relatively moderate injuries can cause symptoms lasting for at least 4 years. This is in accordance with a follow-up study by Taylor et al., where the majority of the subjects showed sequelae 6 months after the initial cold injury, even though most of the injuries were first or second degree (16). Ervasti et al. also conclude that a second degree cold injury to the hands persisted in many cases 4–11 years after the injury (4).

Ten out of 13 patients in the present report, who completed the pain-rating question, reported some degree of pain in the affected hand/foot after 4 months and 7 out of 12 patients reported pain after 4 years. This is consistent with most similar studies where pain is usually listed as one of the most frequent symptoms (5, 6, 7, 8, 16) . However, the pain after 4 months rated low on the 11-grade scale, and had in most cases decreased even more 4 years later. Hence, we consider these findings to be of low clinical value.

The significance of any QST measures are dependent on reliable reference values, in clinical as well as in research settings. In this study, 2 different databases of QST data from healthy subjects were used as reference values (cf. Methods) For all comparisons except for WPT and CPT in the feet, data from other military conscripts were used. The main advantage of this rationale is that the 2 populations were very similar in several measurable aspects. Thus, they were of the same age and the recruiting procedure had been exactly the same for both groups. The main discrepancy between the control group and the tested group was that the procedure for assessing WPT and CPT in the hands differed, with respect to the size of the skin area exposed to the thermal stimuli. In the reference group, the palmar surface of the distal phalange of dig 2 was touching the probe and among the patients, the palmar surface of the distal phalanges of digs 2 *and* 3 were touching the probe. The possible impact of using the dig 2 reference values to compare with the dig 2+3 data is likely to be an underestimation of the measured effects, as the greater skin contact area among the patients would allow faster recognition of temperature changes in the probe than for the reference subjects. Another possible confounding factor may be the use of a second set of reference values, used for WPT and CPT in the feet. This rationale had the advantage that the reference group was tested by the same researcher as conducted the tests in this study, following exactly the same testing procedure, the main disadvantages being the limited number of subjects in the reference group, a greater diversity in age, and a higher mean age. The possible impact from using an older reference group could be an underestimation of the measured effects, since some studies suggest that the ability to detect a change in temperature is reduced with aging (17–20).

One weakness in the study design was the limited accuracy of the description of the severity of the freezing cold injury. Since the data collection was made 4 months post-injury recall bias and the natural healing of the initial injury made more accurate grading of the severity of the freezing cold injury impossible. Therefore, the cold injuries were not classified according the conventional system, from first to fourth degree cold injury, and any relationship between the magnitude of the neurosensory sequelae and the severity of the cold injuries could not be assessed.

Our results confirm the notion that individuals affected by even a moderate freezing cold injury might risk sustained sensory nerve damage. This finding emphasizes the importance of correct handling of situations at which cold exposure cannot be avoided. The findings also underline the importance of a correct acute medical handling of a suspected local freezing cold injury. In clinical occupational medicine, QST is most frequently used to assess patients exposed to hand–arm vibrations. Our findings identify a possible confounder or effect modifier to QST findings previously considered to be caused by vibration exposure alone.

## Conclusion

Neurosensory sequelae after local freezing cold injury, in terms of abnormal thermal and/or vibration perception thresholds, may last at least 4 months after the initial injury. Symptoms such as pain/discomfort at cold exposure, cold sensations and white fingers may persist for at least 4 years after the initial injury.
